# Implementation of a Teledentistry Platform for Dental Emergencies for the Elderly in the Context of the COVID-19 Pandemic in Chile

**DOI:** 10.1155/2022/6889285

**Published:** 2022-03-22

**Authors:** Víctor Beltrán, Alfredo von Marttens, Pablo Acuña-Mardones, Cristina Sanzana-Luengo, Sandra J. Rueda-Velásquez, Eloy Alvarado, Marco Flores, Angeline Cerda, Bernardo Venegas

**Affiliations:** ^1^Clinical Investigation and Dental Innovation Center (CIDIC), Dental School and Center for Translational Medicine (CEMT-BIOREN), Universidad de La Frontera, Temuco 4811230, Chile; ^2^Doctoral Program in Morphological Sciences, Department of Basic Sciences, Faculty of Medicine, Universidad de La Frontera, Temuco 4811230, Chile; ^3^Postgraduate Program in Oral Implantology, Facultad de Odontología, Universidad de Chile, Santiago 8320000, Chile; ^4^Department of Oral Pathology, Faculty of Dentistry, Universidad Santo Tomás, Bucaramanga 680001, Colombia; ^5^Department of Statistics, Faculty of Science, Universidad de Valparaíso, Valparaíso 2340000, Chile; ^6^Dental School, Faculty of Dentistry, Universidad de La Frontera, Temuco 4811230, Chile; ^7^Department of Stomatology, Faculty of Health Sciences, Universidad de Talca, Talca 3460000, Chile; ^8^Carlos Van Buren Hospital of Valparaíso, 2340000, Chile

## Abstract

**Objectives:**

To develop and implement a “semi-presential” technology platform to support urgent and priority dental care for the elderly in the context of the COVID-19 pandemic among the Chilean population.

**Methods:**

A dental mobile clinic was implemented along with the development of a technological platform designed to support emergency and priority dental procedures, including teleconsultation with specialists. Under strict biosafety protocols, dental care was provided in five Chilean regions between February and May 2021. Sociodemographic, medical, and dental data were recorded.

**Results:**

A total of 135 patients over sixty years old, with a mean age of 72 years, were treated, 48 males and 87 females were attended between February and May 2021 in five different regions of Chile. 53.3% required immediate or urgent treatment, and 24.4% were derived to specialists from whom 60.6% needed immediate or urgent treatment. 74.3% of teleconsultations were derived to an oral pathology specialist.

**Conclusion:**

It was shown that a “semi-presential” technology platform implemented in a mobile dental clinic can help elderly people who are impeded to look for traditional dental assistance during a pandemic.

## 1. Introduction

SARS-CoV-2 is transmitted through respiratory route (secretions and aerosols) from symptomatic and asymptomatic infected individuals. Due to its rapid spread, several countries implemented control measures, such as the restriction of mobility and quarantines [1]. Due to the nature of the dental practice, it is necessary to establish strict biosafety protocols to prevent cross infection, especially when dental procedures that potentially generate aerosols are performed. Attention has been limited to priority consultations or dental emergencies [2]. Thus, both the fear of contagion by leaving their homes and a higher mortality risk for elderly people due to their intrinsic vulnerability lead to a lack of proper attention to several dental conditions, compromising their health and quality of life [3]. In fact, dental infections and emergencies rose considerably in the context of the COVID-19 pandemic [4].

Currently, the COVID-19 pandemic has increased the challenges related to the provision of health services to the elderly population, considering these services are institutionalized or not. The need of stronger public policies, more economic resources and qualified personnel became evident in order to attend the complex necessities of this age group that require greater attention with emphasis in oral health [5]. Late oral cancer diagnosis is a frequent problem in Chile, and the difficulty to normally attend to controls could increase this severe cause of death among the elderly [6]. A great deal of innovation has been required in dental attention protocols to avoid neglecting proper dental health attention and to continue providing adequate services to this vulnerable population. Teledentistry has gained momentum and validity during the present pandemic, becoming a powerful tool which, through the use of technology, offers solutions that allow for continuous attention, reaching diagnosis and providing online or remote treatment, also facilitating the education and orientation of patients in oral health issues. All of this contributes to avoiding the risk of infection by keeping the patients away from crowded high-risk areas such as dental offices and hospitals [7] and also allows for the early detection of lesions that may result in oral cancer.

Bearing this situation in mind, a novel semipresential platform to support urgent and priority dental attention for elderly people in the context of the COVID-19 pandemic was developed and deployed by our research team. The platform allowed for real-time or asynchronous interaction between the patient and a team of specialists that support the attention provided by a general dentist in a mobile dental clinic with digital equipment that can move close to or right in front of the patient's place of residence, permitting prompt diagnosis and providing care to elderly people who were confined to their places of residence due to the implemented lockdown measures at that time. In Chile, as in many other parts of the world, there has been an increase in elderly population due to lower mortality and birth rates [8]. In fact, it is estimated that more than 30% of the population will be older than 60 by 2050 [9]. Chile is also the only country in Latin America with a life expectancy of over 80 years old. The increase of oral cancer cases might be derived from this higher life expectancy, and a late diagnostic can be associated to the pandemic, due to the impossibility of a normal presential clinical evaluation. This pilot study not only focuses on the resolution of current dental health challenges but also focuses on the generation of attention protocols through teledentistry that would enhance future dental care coverage for the elderly population and contribute to the early detection of possible malignant lesions.

The main objective of this study was to develop and deploy a technological semipresential platform to support emergency and priority dental attention for elderly patients in the context of the COVID-19 pandemic in the Chilean population. With that purpose, we developed and deployed a pilot teledentistry program using a digital/technological platform that would allow for an online or remote interaction between a patient and a multidisciplinary team of dental specialists and geriatricians, who would provide support to a general dentist onboard a mobile clinic in the resolution of dental emergencies in five regions of Chile.

## 2. Materials and Methods

Between June 2020 and January 2021, a web-based platform in conjunction with a mobile app for teledentistry was specially designed for the attention of the elderly population aiming to facilitate the recording of medical and dental anamnesis and to allow for interconsultation with different dental specialists and geriatricians. This web platform was implemented in a mobile dental clinic to provide dental care to elderly patients in 5 regions of Chile between February and May 2021. This platform was advertised to the elderly patients through seminar sessions given to personnel from the National Service for the Elderly (SENAMA-Chile). The study was developed as part of the project “Semi-presential technological platform to support urgent and priority dental care for the elderly in the context of the COVID-19 pandemic in the Chilean population” supported by the Ministry of Science, Technology, Knowledge and Innovation of Chile through the National Agency of Research and Development (ANID). Ethical approval for carrying out this project was granted by the Universidad de la Frontera Ethics Committee, decision 090/20 (Project ANID 0766 2020, National Research and Development Agency).

### 2.1. The Platform Concept and Architecture

**Figure 1:**
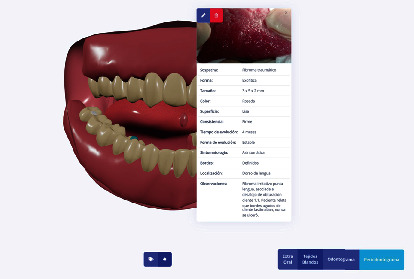
Platform module interface, 3D standardized model, labeled with a presumptive diagnosis (intraoral examination, soft tissues)

A conceptual design of the web-based platform was made that included a visualization of process-level interfaces and workflows. The design of the technological architecture included the definition of the components of the system, interface language (Spanish), and technologies, along with the construction of the software modules with their respective functional verification. Each of the stages was approached following an iterative and incremental methodology, based on good software development practices, that ensured the control, verification, and quality of software development. The software interface was developed in a modular design and includes anamnesis modules and a novel 3D standardized model for indexing relevant information for each case (Figure 1). A specially designed digital representation system for oral lesions was included in the platform design, allowing for a proper visualization by the specialist in order to provide an accurate diagnosis.

### 2.2. Patients

A sample calculation was performed considering a minimum number of patients to provide dental assistance in the context of the project, this sample being representative of the Chilean population. The allocation for five regions was determined proportionally to the resident population according to the projections made by the National Statistics Institute (INE) of Chile. These regions were considered representative due to their location in the north (Antofagasta), center (Santiago and Talca), and south of Chile (Concepción and Temuco). A number of complementary patients were considered, in order to ensure the number of patients required by location established by the study. Descriptive statistical analysis was performed with R-project, 4.0.3 version.

**Figure 2:**
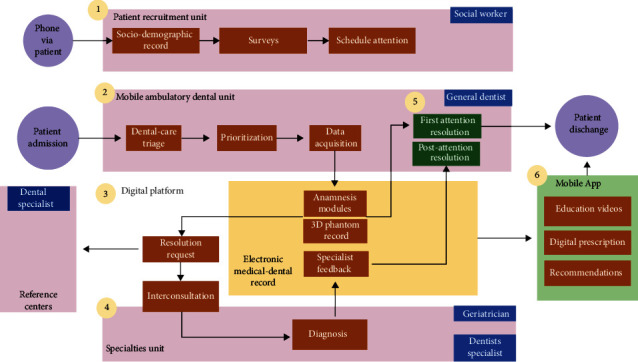
Operational schematics of the attention and platform use workflow to support dental-medical care for the elderly in the context of the COVID-19 pandemic. Pink boxes: macroprocess or units; green boxes: software; blue boxes: professional in charge of the macroprocess or unit; orange boxes: subprocess.

Inclusion criteria considered elderly population (over 60 years) with a dental emergency or requiring some kind of priority dental care. Requirements to provide dental care in a mobile clinic were specified as follows: sufficient mobility to access a dental chair in a mobile clinic; the patient and/or their caregiver must be able to handle a smartphone or some other electronic device connected to the Internet that allows for an application (an App for Android devices) to be downloaded and installed; patients with chronic diseases must be under pharmacological treatment according to medical indications; patients must be capable of receiving verbal instructions and must complete a triage prior to the provision of dental care. The professional team who operated the system underwent proper training. An operational schematic of the attention and platform use workflow for the development of the study is shown in Figure 2.

Patients were enrolled by specialized personnel (social workers) from the National Service for the Elderly (SENAMA-Chile). Data considered for the patient's registration included their full name, national ID number, date of birth, contact phone, address, and also their caregiver or close family member's full name and contact phone number. Patient confidentiality was granted by anonymization of personal information prior to data treatment.

A Dental Care Triage was performed, considering that any of the following conditions or more than one had to be present: severe dental pain refractory to analgesic therapy; recent trauma (direct blow involving teeth or mouth, accompanied by severe pain); oral bleeding; significant swelling of any anatomical part of the mouth, face, or neck; pigmented lesions or wounds in any part of the mouth that had not disappeared in a month; loss or fracture of restorations (fillings) or dental prostheses; and injuries to the oral mucosa due to dental prosthesis mismatch and dental treatment required prior to critical medical procedures that cannot be postponed (e.g., patient who would undergo radiotherapy requiring a previous tooth extraction). Infographic material was used to facilitate the patient's understanding. Patients who were users of anticoagulant drugs had chronic diseases without treatment or were in antineoplastic treatment or dialysis were excluded, unless a medical indication to be treated was provided.

Type of dental treatment required was classified as (1) no treatment needed, (2) need for dental cleaning, (3) nonimmediate treatment needed, (4) immediate treatment needed, and (5) urgent care needed [10]. Interconsultations were electronically sent to specialists in oral pathology, periodontology, oral rehabilitation, oral imaging, temporomandibular joint disorders, and geriatrics. The specialist provided an answer on the same platform either synchronously or asynchronously. More complex procedures that could not be provided in situ, such as surgical biopsies, were performed by specialists (also members of the project team) in external reference centers. Postdischarge follow-up was performed through an Android mobile app to be installed on the patient's or caretaker's smartphone. Customized information, including educational videos and dental recommendations, was sent to each patient or caretaker through this app after obtaining their informed consent.

## 3. Results

**Figure 3:**
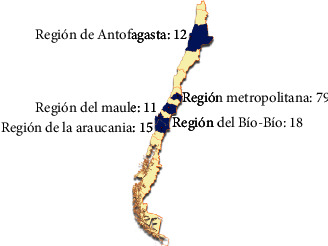
Map showing selected regions of Chile for the study. Patients enrolled and treated between February and May 2021.

The target population of this study was senior Chilean citizens living in the regions of Antofagasta, Metropolitana, Maule, Bío-Bío, and Araucanía, given the feasibility of implementing the teledentistry platform in these geographical areas. Nonetheless, within each region, we followed a probabilistic sampling design to select which districts were to be included in the study. Given financial constraints, we used a sample size of 135 which were allocated using a proportional allocation with respect to the region population size.  Figure 3 shows the selected districts within each region and their respective allocations that are as follows: 79 patients from the Maipú district in Región Metropolitana, 18 patients from the Concepción district in Región del Bío-Bío, 15 patients from the Temuco district in Región de la Araucanía, 12 patients from the Antofagasta district in Región de Antofagasta, and 11 patients from the Talca district in Región del Maule.

Data was collected from patients by the general dentist and a dental assistant. Information was recorded on the platform that was named Geriatric Dental Specialties Teleplatform (TEGO by its acronym in Spanish: “Teleplataforma de Especialidades Geriátrico Odontológicas”). Recorded data included sociodemographic variables (gender and age), work status (retired and not retired), health plan (FONASA, ISAPRE, or CAPREDENA), residence status (living alone, with family, or in a senior residence), and the vulnerability level (lowest 40%, between 40% and 60%, 60% and higher). FONASA is a public National Health Fund, ISAPRE corresponds to private social security institutions, and CAPREDENA is a military national defence provident fund. The vulnerability level is measured through the “Household Social Registry,” which is a database that has all the necessary information of people and households to support the process of application and selection of the beneficiaries of the state institutions in Chile.

In this study, 135 patients were treated in the context of the project, with a mean age of 72 years. Number of patients was defined mainly considering available resources from the project and distributed according to a proportional calculation given the total population of the selected region. COVID-19 security protocols were used following instructions from the Chilean Ministry of Health. 48 patients were male and 87 were female. As seen in Table 1, most patients included in this study live in the capital region due to the proportional allocation used (58.52% of patients). As for work status and health plan status, most patients were retired (75.56%) and had a national health plan fund (FONASA, 97.03%). As for residence status, most patients lived with either family members or in a senior living facility (72.59%). Finally, most patients belonged to the second quintile of the population according to income (77.03%), this categorization plays an important role in Chilean public policies as it is a necessary condition to receive certain governmental benefits [11].

To determine the type of dental care provided to the patients (urgent or priority dental care), the following categories were used: dental cleaning needed (3 patients), no immediate dental treatment needed (60 patients), immediate dental treatment needed (36 patients), and urgent treatment needed (36 patients). 33 patients needed interconsultation to different specialties.

In Table 1, a brief statistical summary of the patients involved in this study and the type of dental care needed have been provided.  Table 2 shows the frequency of interconsultation by specialty and region, with 26 interconsultations sent to oral pathology, 5 to geriatrics, 2 to temporomandibular joint disorders, 1 to oral rehabilitation, and 1 to oral radiology.  Table 3 shows the frequency of interconsultation by urgency and region. Among oral pathology interconsultations, 12 reactive lesions (fibroma, frictional keratosis, mucocele), 5 infectious lesions (subprosthetic stomatitis, median rhomboid glossitis, chronic periodontitis), 4 vascular etiology lesions (vascular malformation), 3 pigmented lesions (amalgam tattoo and smoking-related melanosis), and 3 potentially malignant lesions (leukoplakia, erythroplakia, and lichen planus) were diagnosed. Details are shown in Table 4.

## 4. Discussion

The results of this study have shown that teledentistry using a web platform specially designed to allow for the remote interaction between a general dentist attending elderly patients and a staff of different dental specialities and geriatricians is an excellent alternative to provide dental care for this population that is particularly vulnerable and frail. Dental care for the elderly in Chile is provided mainly by the public health system [8]. Several conditions limit this activity due to the high demand of this type of care, poor state of oral health, and the reduced possibility of offering sufficient coverage for the elderly. The COVID-19 pandemic brought an additional difficulty due to the existence of a strict government plan of confinement that included extensive lockdowns and the reduction of mobility in an attempt to avoid or diminish the risk of contagion among the population, even forbidding the functioning of dental clinics in some regions of the country by local sanitary authorities, which happened for extended periods of time in the Región de la Araucanía, in the south of Chile for example, which generated a lot of controversy among the community of dental professionals in the country [12, 13, 14].

This study has shown that it is possible to bring dental care for the elderly to the place where they reside and provide appropriate dental assistance in a secure manner using a mobile dental clinic equipped with a modern technological platform specially designed for this purpose. Another possible application for this type of platform and mobile unit could be dental care for adults with tetraplegia or other neurodegenerative or neuromotor conditions. A few studies aimed at the home care of this population through the use of telemedicine technologies, similar to what has been proposed in this study [15, 16].

The application of teledentistry might be favoured by a collaborative setting between health, information technology, and education sciences, facilitating the generation of innovative solutions that can turn into technologies with a more efficient social impact [17]. The irregular Chilean geography and its unequal distribution of specialized dental services for elderly people in urban and rural areas bring a persistent difficulty in access possibilities to centers of specialized dental care, which is intrinsically deficient for this population [8]. For older citizens, who live in senior living facilities in Chile, limitations in mobility and lack of staff and vehicles to aid in their transportation to health centers add to this problem. Thus, the implementation of home care with the assistance of technology and mobile clinics is a viable tool for the dental care of people who, due to their vulnerability, must avoid leaving their places of residence or having unnecessary appointments with health professionals, but can still opt for a prompt and adequate care [18].

It is interesting to note that 26.6 % of the patients were categorized as urgent treatment needed. The solution given to these patients contributed to at least partially recovering a certain state of wellness in terms of oral health that could not have occurred without this intervention. Additionally, a similar percentage of patients was categorized as immediate need, although not urgent. This analysis concluded that more than fifty percent of patients required dental treatment in a priority fashion, whose problem would have persisted without this intervention, considering that 97% of the patients are insured by FONASA, the public National Health Fund, and that 75.5% of the patients were retired.

Loneliness is a situation frequently observed among elderly people in Chile [19]. A contribution to psychological wellbeing could be perceived among patients who received attention during the study. The fact of visiting the patient's place of residence itself to provide the assistance implied an act that was well valued by them, also taking in consideration that 27.4% lived alone and 77% belonged to the most vulnerable group of the population, with the lowest incomes.

Besides caries and periodontal disease, oral pathologies are frequent in elderly people [20]. Interconsultations were well valued by patients since they provided prompt solutions to their ailments. In this study, 35 interconsultations were performed by specialists, 26 of them by the oral pathologist. Considering the risk of oral cancer among the elderly people, this is an important factor as a probable method to achieve an early oral cancer diagnosis, aimed at a more preventive than palliative approach [21, 22]. An important percentage of potentially malignant oral lesions and oral cancer might be studied through methods of telediagnosis, aided by real-time or asynchronous online communication between the treating dentist onboard the mobile clinic and the pathologist working remotely through the web-based platform. In cases in which it was necessary, a biopsy study was arranged in some of the referral university centers according to the region/district in which the patient resided. As a main conclusion, it has been demonstrated that a mobile dental care unit is an appropriate solution to provide care for older adults who are prevented from looking for dental assistance in traditional health centers. Furthermore, considering the current COVID-19 pandemic and potential new outbreaks, it is also a good alternative care option to diminish the risk of contagion by providing assistance at the patient's place of residence. It can also be concluded that the technological platform deployed for the study contributed to the early diagnosis of risk pathologies in confined patients such as oral cancer in the context of the COVID-19 pandemic. It can be suggested that the implementation of this kind of dental assistance in a permanent way can be of great significance in contributing to good oral health and the well-being of the elderly. The implementation of technological platforms and apps installed on the users' mobile devices monitoring the oral health of elderly people might also be recommended, since it permits the education of their caretakers and the training of health care staff, also optimizing the dental coverage of specialist care.

## 5. Conclusion

It was shown that a “semi-presential” technology platform implemented in a mobile dental clinic can help elderly people who are impeded to look for traditional dental assistance during a pandemic. It contributes to reduce the risk of contagion, and it could be of great significance as a permanent solution to improve the oral health and general quality of life of the elderly population. It also contributes to the early diagnosis of risk pathologies such as potentially malignant disorders, thus preventing oral cancer occurrence.

## Figures and Tables

**Table 1:** Summary of sociodemographic variables of patients involved in this study by region. Standard deviation of age is provided in parenthesis. ∗The lower the percentage, the higher the level of vulnerability.

Sociodemographic variables	*n*	Bío-Bío	Araucanía	Maule	Metropolitana	Antofagasta
Mean age (years)		70.67 (5.28)	69.87 (8.49)	71.55 (6.33)	72.67 (6.34)	70.84 (5.42)
Gender						
Male	48	9	8	6	23	2
Female	87	9	7	5	56	10
Pension						
Retired	102	15	11	9	56	11
Not retired	33	3	4	2	23	1
Health plan						
FONASA	131	18	15	11	75	12
ISAPRE	2				2	
CAPREDENA	2				2	
Residence status						
Living alone	37	1	5	1	30	
Living with family	88	16	10	1	49	12
Senior residence	10	1		9		
Vulnerability level						
Lowest 40%∗	104	14	13	11	60	6
Between 40% and 60%	23	2	2	0	13	6
60% and higher	8	2			6	

**Table 2:** Frequency of interconsultation by specialty and region of Chile.

Medical specialities	*n*	Bío-Bío	Araucanía	Maule	Metropolitana	Antofagasta
Oral pathology	26	4	5	2	14	1
Oral rehabilitation	1	1				
Geriatrics	5		1		3	1
Oral radiology	1				1	
Temporomandibular joint disorders	2				2	

**Table 3:** Frequency of interconsultation by urgency and region of Chile.

Urgency	*n*	Bío-Bío	Araucanía	Maule	Metropolitana	Antofagasta
Dental cleaning needed						
Interconsultation	1				1	
No interconsultation	2		1		1	
No immediate treatment need						
Interconsultation	12	4	1		5	2
No interconsultation	48	7	6	4	25	6
Immediate treatment need						
Interconsultation	8			1	7	
No interconsultation	28	3		5	18	2
Urgent treatment needed						
Interconsultation	12	1	5	1	5	
No interconsultation	24	3	2		17	2

**Table 4:** Age, sex, pathology groups, and localization of 27 oral lesion interconsultations (*n* = 26).

Variable		
Age		Mean (SD)
		72 (6.5)
Sex		*n* (%)
	Men	11 (42)
	Women	15 (58)
Pathology	Reactive lesions	12
	Infectious lesions	5
	Vascular lesions	4
	Pigmented lesions	3
	Potentially malignant lesions	3
		27
Localization	Vermilion	2
	Labial mucosa	4
	Jugal mucosa	5
	Palate	2
	Gums	5
	Tongue	8
		26

## Data Availability

The data used to support the findings of this study are included within the article.
